# Psychosis in Systemic Lupus Erythematosus: Results From an International Inception Cohort Study

**DOI:** 10.1002/art.40764

**Published:** 2019-01-18

**Authors:** John G. Hanly, Qiuju Li, Li Su, Murray B. Urowitz, Caroline Gordon, Sang‐Cheol Bae, Juanita Romero‐Diaz, Jorge Sanchez‐Guerrero, Sasha Bernatsky, Ann E. Clarke, Daniel J. Wallace, David A. Isenberg, Anisur Rahman, Joan T. Merrill, Paul R. Fortin, Dafna D. Gladman, Ian N. Bruce, Michelle Petri, Ellen M. Ginzler, M. A. Dooley, Kristjan Steinsson, Rosalind Ramsey‐Goldman, Asad A. Zoma, Susan Manzi, Ola Nived, Andreas Jonsen, Munther A. Khamashta, Graciela S. Alarcón, Ronald F. van Vollenhoven, Cynthia Aranow, Meggan Mackay, Guillermo Ruiz‐Irastorza, Manuel Ramos‐Casals, S. Sam Lim, Murat Inanc, Kenneth C. Kalunian, Soren Jacobsen, Christine A. Peschken, Diane L. Kamen, Anca Askanase, Chris Theriault, Vernon Farewell

**Affiliations:** ^1^ Queen Elizabeth II Health Sciences Center and Dalhousie University Halifax Nova Scotia Canada; ^2^ University of Cambridge Cambridge UK; ^3^ Toronto Western Hospital and University of Toronto Toronto Ontario Canada; ^4^ University of Birmingham Birmingham UK; ^5^ Hanyang University Hospital for Rheumatic Diseases Seoul Republic of Korea; ^6^ Instituto Nacional de Ciencias Medicas y Nutrición Mexico City Mexico; ^7^ McGill University Montreal Quebec Canada; ^8^ University of Calgary Calgary Alberta Canada; ^9^ Cedars‐Sinai Medical Center and David Geffen School of Medicine at University of California Los Angeles; ^10^ University College London London UK; ^11^ Oklahoma Medical Research Foundation Oklahoma City; ^12^ CHU de Québec Université Laval Quebec City Quebec Canada; ^13^ University of Manchester and Manchester University NHS Foundation Trust Manchester UK; ^14^ Johns Hopkins University School of Medicine Baltimore Maryland; ^15^ SUNY Downstate Medical Center Brooklyn New York; ^16^ University of North Carolina Chapel Hill; ^17^ Landspitali University Hospital Reykjavik Iceland; ^18^ Northwestern University Chicago Illinois; ^19^ Hairmyres Hospital East Kilbride Scotland UK; ^20^ Lupus Center of Excellence, Allegheny Health Network Pittsburgh Pennsylvania; ^21^ Lund University Lund Sweden; ^22^ St Thomas’ Hospital King’s College London School of Medicine London UK; ^23^ University of Alabama at Birmingham; ^24^ Karolinska Institute Stockholm Sweden; ^25^ Feinstein Institute for Medical Research Manhasset New York; ^26^ Hospital Universitario Cruces University of the Basque Country Barakaldo Spain; ^27^ Hospital Clínic Barcelona Spain; ^28^ Emory University School of Medicine Atlanta Georgia; ^29^ Istanbul University Istanbul Turkey; ^30^ University of California San Diego School of Medicine La Jolla; ^31^ Rigshospitalet, Copenhagen University Hospital Copenhagen Denmark; ^32^ University of Manitoba Winnipeg Manitoba Canada; ^33^ Medical University of South Carolina Charleston; ^34^ Hospital for Joint Diseases, New York University New York New York

## Abstract

**Objective:**

To determine, in a large, multiethnic/multiracial, prospective inception cohort of patients with systemic lupus erythematosus (SLE), the frequency, attribution, clinical, and autoantibody associations with lupus psychosis and the short‐ and long‐term outcomes as assessed by physicians and patients.

**Methods:**

Patients were evaluated annually for 19 neuropsychiatric (NP) events including psychosis. Scores on the Systemic Lupus Erythematosus Disease Activity Index 2000, the Systemic Lupus International Collaborating Clinics/American College of Rheumatology Damage Index, and the Short Form 36 (SF‐36) were recorded. Time to event and linear regressions were used as appropriate.

**Results:**

Of 1,826 SLE patients, 88.8% were female and 48.8% were Caucasian. The mean ± SD age was 35.1 ± 13.3 years, the mean ± SD disease duration was 5.6 ± 4.2 months, and the mean ± SD follow‐up period was 7.4 ± 4.5 years. There were 31 psychotic events in 28 of 1,826 patients (1.53%), and most patients had a single event (26 of 28 [93%]). In the majority of patients (20 of 25 [80%]) and events (28 of 31 [90%]), psychosis was attributed to SLE, usually either in the year prior to or within 3 years of SLE diagnosis. Positive associations (hazard ratios [HRs] and 95% confidence intervals [95% CIs]) with lupus psychosis were previous SLE NP events (HR 3.59 [95% CI 1.16–11.14]), male sex (HR 3.0 [95% CI 1.20–7.50]), younger age at SLE diagnosis (per 10 years) (HR 1.45 [95% CI 1.01–2.07]), and African ancestry (HR 4.59 [95% CI 1.79–11.76]). By physician assessment, most psychotic events resolved by the second annual visit following onset, in parallel with an improvement in patient‐reported SF‐36 summary and subscale scores.

**Conclusion:**

Psychosis is an infrequent manifestation of NPSLE. Generally, it occurs early after SLE onset and has a significant negative impact on health status. As determined by patient and physician report, the short‐ and long‐term outlooks are good for most patients, although careful follow‐up is required.

## Introduction

Neuropsychiatric (NP) events are one of the features of systemic lupus erythematosus (SLE), but their frequency and attribution to SLE or other causes is variable. Overall, approximately one‐third are caused directly by SLE 1, but for individual manifestations this varies between 0% and 100% 2, 3. The outcome for individual NPSLE manifestations, especially rare NP events, is derived from observational cohorts of well‐characterized patients followed up over prolonged periods.

One of the rarer NP events is lupus psychosis, which is part of both the American College of Rheumatology (ACR) 4 and the Systemic Lupus International Collaborating Clinics (SLICC) 5 classification criteria for SLE. Characterized by delusions and hallucinations, it is a dramatic presentation of NPSLE 6, 7. It is one of the few manifestations of nervous system disease in SLE associated, although inconsistently, with a lupus‐specific autoantibody against ribosomal P 8, 9, 10. The infrequent occurrence of psychosis has limited the number of clinical studies, and most consist of case series obtained by review of medical records. In the present study of lupus psychosis, we determined its frequency, attribution, clinical, and autoantibody associations and the outcome assessed by physicians and patients in a large, multiethnic/multiracial, prospective inception cohort of SLE patients.

## Patients and Methods

### Research study network

The study was conducted by the SLICC 11, a network of 53 investigators in 43 academic medical centers in 16 countries. The current study involved 31 centers in 10 countries. Data were collected per protocol at enrollment and annually, submitted to the coordinating center in Halifax, Nova Scotia, Canada, and entered into an Access database. Appropriate procedures ensured data quality, management, and security. The Nova Scotia Health Authority central zone Research Ethics Board, Halifax, and each of the participating centers’ institutional research ethics review boards approved the study.

### Patients

Patients fulfilled the ACR classification criteria for SLE 4, which served as the date of diagnosis, and provided written informed consent. Enrollment was permitted up to 15 months following the diagnosis. Demographic variables, education, and medication history were recorded. Lupus‐related variables included the Systemic Lupus Erythematosus Disease Activity Index 2000 (SLEDAI‐2K) 12 and the SLICC/ACR Damage Index (SDI) 13. Laboratory testing required to determine the SLEDAI‐2K and SDI scores was done at each center.

### NP events

An enrollment window extended from 6 months prior to the diagnosis of SLE up to the actual enrollment date. NP events were characterized within this window using the ACR case definitions for 19 NP syndromes 14. The clinical diagnosis was supported by investigations, if warranted, as per the guidelines. Patients were reviewed annually within a 6‐month window around the assessment date. New NP events and the status of previous NP events since the last study visit were determined at each assessment.

The ACR case definition for psychosis 14 includes the following: 1) delusions or hallucinations without insight; 2) causing clinical distress or impairment in social, occupational, or other relevant areas of functioning; 3) disturbance should not occur exclusively during delirium; and 4) not better accounted for by another mental disorder. Recurring episodes of psychosis and other NP events within the enrollment window or within a follow‐up assessment period were recorded once for that period of observation. The date of the first episode was taken as the onset of the event. Once an NP event had resolved, a subsequent event of the same type was recorded as a new event.

### Attribution of NP events

Decision rules used to determine the attribution of NP events were similar to those reported in other publications concerning the SLICC NPSLE inception cohort 15, 16. We considered 3 factors. The first was the temporal onset of NP event(s) in relation to the diagnosis of SLE, and the second concerned concurrent non‐SLE factor(s), such as potential causes (“exclusions”) or contributing factors (“associations”) for each NP syndrome in the glossary for the ACR case definitions of NP events 14. For psychosis, the prespecified potential alternative causes (exclusions) were 1) primary psychotic disorder unrelated to SLE (e.g., schizophrenia), 2) substance‐ or drug‐induced psychotic disorder, and 3) psychologically mediated reaction to SLE (brief reactive psychosis with major stressor); the prespecified potential contributing factors (associations) were 1) marked psychosocial stress and 2) corticosteroids. For the third and final factor, we identified “common” NP events in normal population controls as described by Ainiala et al 17. These included isolated headaches, anxiety, and mild depression (mood disorders failing to meet criteria for “major depressive‐like episodes”), mild cognitive impairment (deficits in less than 3 of the 8 specified cognitive domains), and polyneuropathy without electrophysiologic confirmation. Using these 3 factors, we used 2 attribution decision rules of different stringency (models A and B) 15, 16.

#### Attribution model A (most stringent)

NP events that had their onset within the enrollment window *and* had no exclusions or associations *and* were not one of the NP events identified by Ainiala et al 17 were attributed to SLE.

#### Attribution model B (least stringent)

NP events that had their onset within 10 years of the diagnosis of SLE and were still present within the enrollment window *and* had no exclusions *and* were not one of the NP events identified by Ainiala et al 17 were attributed to SLE.

By definition, all NP events attributed to SLE using model A were similarly attributed using model B. Events that did not fulfill these criteria were classified as non‐SLE NP events.

### Outcome of psychosis

For every NP event, a physician‐generated 7‐point Likert scale was completed at each follow‐up assessment until resolution of the event or patient demise (1 = patient demise, 2 = much worse, 3 = worse, 4 = no change, 5 = improved, 6 = much improved, 7 = resolved) 18. A patient‐generated Short Form 36 (SF‐36) questionnaire was also completed at each assessment and provided subscale scores, mental component summary (MCS) and physical component summary (PCS) scores 18, 19 that were unavailable to physicians at their assessments.

### Autoantibodies

Plasma lupus anticoagulant, serum IgG anticardiolipin, anti–β_2_‐glycoprotein I, anti–ribosomal P (anti‐P), and anti–NR2 glutamate receptor antibodies were measured at the Oklahoma Medical Research Foundation, as described 20, 21, 22, 23.

### Statistical analysis

Since there were only 15 patients with psychosis attributed to SLE by model A, we used attribution model B and Cox regression to analyze time to first SLE psychosis. This included onset of NP events prior to SLE diagnosis in order to capture all NP events potentially related to the risk of psychosis.

Hazard ratios (HRs) and 95% confidence intervals (95% CIs) were calculated. Covariates examined included sex, race/ethnicity, SLICC sites, postsecondary education, number of ACR criteria at enrollment (excluding neurologic disorder), SDI (without NP variables), other concurrent NP events, and, as continuous variables, age at SLE diagnosis, disease duration (in years), and SLEDAI‐2K score (without NP variables). Binary variables indicating autoantibodies present at baseline and follow‐up assessments were defined when available. Time‐varying variables, other than those related to autoantibodies, were updated at each assessment. When examining the time‐varying version of the autoantibody variables, autoantibody data in the period before enrollment were imputed by their values at enrollment, while autoantibody data at follow‐up assessments were imputed by the last observation carried forward method. Kaplan‐Meier estimates of the survivor function for the time until resolution of psychosis were calculated. For analyses of longitudinal SF‐36 subscale and summary scores, linear regression with generalized estimating equations allowed for correlation of observations within patients, and adjustment variables included time/visit, sex, age at SLE diagnosis, race/ethnicity/location, education, SLEDAI‐2K and SDI scores (without NP variables), corticosteroids, antimalarials, and immunosuppressant use since last assessment.

## Results

### Patient recruitment and assessments

A total of 1,826 patients were recruited between October 1999 and December 2011, from centers in the US (n = 539 [29.5%]), Europe (n = 477 [26.1%]), Canada (n = 418 [22.9%]), Mexico (n = 223 [12.2%]), and Asia (n = 169 [9.3%]) (Table 1). The number of patient assessments varied from 1 to 19 with a mean ± SD follow‐up period of 7.4 ± 4.5 years, and final assessment follow‐up was in March 2017.

**Table 1:** Demographics, clinical features, medications, and autoantibodies in the 1,826 patients at enrollment^a^

Sex, no. (%)	
Female	1,622 (88.8)
Male	204 (11.2)
Age, years	35.1 ± 13.3
Race/ethnicity, no. (%)	
Caucasian	891 (48.8)
African	306 (16.8)
Hispanic	282 (15.4)
Asian	275 (15.1)
Other	72 (3.9)
Marital status, no. (%)	
Single	818 (44.9)
Married	766 (42.0)
Other	238 (13.1)
Postsecondary education, no. (%)	1,064 (61.9)
Disease duration, months	5.6 ± 4.2
ACR SLE criteria met	4.9 ± 1.1
ACR SLE criteria, no. (%)	
Malar rash	660 (36.1)
Discoid rash	227 (12.4)
Photosensitivity	652 (35.7)
Oral/nasal ulcers	677 (37.1)
Serositis	502 (27.5)
Arthritis	1,368 (74.9)
Renal disorder	510 (27.9)
Neurologic disorder	88 (4.8)
Hematologic disorder	1,129 (61.8)
Immunologic disorder	1,392 (76.2)
Antinuclear antibody positivity	1,731 (94.8)
SLEDAI‐2K score	5.3 ± 5.4
SLICC/ACR Damage Index score^b^	0.32 ± 0.74
Medications, no. (%)	
Corticosteroids	1,284 (70.3)
Antimalarials	1,231 (67.4)
Immunosuppressants	732 (40.1)
ASA	261 (14.3)
Antidepressants	183 (10.0)
Warfarin	99 (5.4)
Anticonvulsants	80 (4.4)
Antipsychotics	12 (0.7)
Autoantibody positivity, no./total no. (%)	
Lupus anticoagulant	241/1,174 (20.5)
Anticardiolipin	138/1,142 (12.1)
Anti–β_2_‐glycoprotein I	163/1,142 (14.3)
Anti–ribosomal P	112/1,136 (9.9)
Anti–NR2 glutamate receptor	130/1,064 (12.2)

a Except where indicated otherwise, values are the mean ± SD. SLEDAI‐2K = Systemic Lupus Erythematosus Disease Activity Index 2000; ASA = acetylsalicylic acid.

b The Systemic Lupus International Collaborating Clinics/American College of Rheumatology (SLICC/ACR) Damage Index score was not available for 1,057 patients at the enrollment visit when disease duration was <6 months.

### NP manifestations

NP events (≥1) occurred in 951 of 1,826 patients (52.1%), and 488 of 1,826 patients (26.7%) had ≥2 events during the study period. There were 1,902 unique NP events, encompassing all 19 NP syndromes in the ACR case definitions 14. The proportion of NP events attributed to SLE varied from 17.8% (attribution model A) to 31.1% (attribution model B) and occurred in 13.3% of patients (model A) to 21.1% of patients (model B). Of the 1,902 unique NP events, 1,742 (91.6%) involved the central nervous system and 160 (8.4%) involved the peripheral nervous system 14. The classification of events as diffuse and focal was 1,471 (77.3%) and 431 (22.7%), respectively 16.

### Psychosis

**Figure 1:**
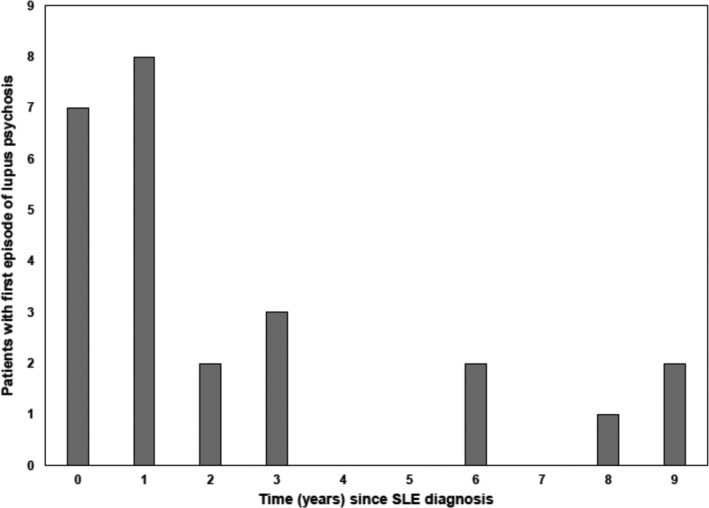
Relationship between the time of onset of lupus psychosis and diagnosis of systemic lupus erythematosus (SLE).

Among 28 of 1,826 patients with psychosis (1.53%), 26 of 28 (93%) had a single psychotic event, while 1 patient had 2 discrete events and 1 patient had 3 discrete events. The majority of patients had psychosis attributed to SLE (15 of 28 [54%] using attribution model A and 25 of 28 [89%] using attribution model B). Patients with lupus psychosis (model B) were located in centers in Europe (9 patients), Canada (6 patients), the US (5 patients), Mexico (4 patients), and Asia (1 patient). There was no significant association between location and risk of SLE psychosis (*P* = 0.53 in Cox regression) taking into account the number of patients and the duration of follow‐up at each site. The majority of patients with lupus psychosis (20 of 25 [80%]) had their first episode either in the year prior to or within 3 years following the diagnosis of SLE (Figure 1). There were 31 psychotic events, of which 16 of 31 (52%) and 28 of 31 (90%) were attributed to SLE using attribution models A and B, respectively. The earliest psychotic episode occurred 2 months prior to the diagnosis of SLE.

### Clinical and laboratory associations with lupus psychosis

Using Cox regression, we looked for associations with the risk of the first episode of psychosis attributed to SLE using attribution model B. Univariate analysis revealed positive associations with male sex (HR 2.58 [95% CI 1.04–6.41]), younger age at diagnosis (per 10 years) (HR 1.36 [95% CI 1.0–1.88]), African ancestry (HR 4.80 [95% CI 1.86–12.40]), in particular for patients outside the US (HR 5.53 [95% CI 1.86–16.42]), concurrent other central (HR 3.86 [95% CI 1.27–11.70]) or diffuse (HR 6.36 [95% CI 2.12–19.12]) NP events (mood disorder, acute confusional state) attributed to SLE, and presence of anti‐P antibodies at the enrollment visit into the cohort (HR 3.31 [95% CI 1.19–9.21]) and over time (HR 3.13 [95% CI 1.15–8.56]).

Important variables identified in univariate analyses, in particular for African patients outside the US, were included in multivariate analyses, excluding antibody variables due to reduced sample size consequent to missing data (Table 2). The significant positive associations with lupus psychosis were similar, namely, prior SLE NP events (HR 3.59 [95% CI 1.16–11.14]), male sex (HR 3.0 [95% CI 1.20–7.50]), younger age at SLE diagnosis (per 10 years) (HR 1.45 [95% CI 1.01–2.07]), and African ancestry (HR 4.59 [95% CI 1.79–11.76]). Further, after adjustment for the demographic predictors in Table 2 (sex, age at SLE diagnosis, and race/ethnicity), anti‐P antibodies at enrollment (HR 2.29 [95% CI 0.81–6.46], *P* = 0.11) and over time (HR 2.17 [95% CI 0.79–5.97], *P* = 0.13) were no longer significantly associated with the risk of lupus psychosis.

**Table 2:** Predictors of lupus psychosis by multivariate analysis^a^

Predictor, factor level	HR (95% CI)	*P*
Other concurrent NP events		
No concurrent NP events	1	
Any unresolved NP events attributed to SLE	3.59 (1.16–11.14)	0.027^b^
Any unresolved NP events not attributed to SLE but no events attributable to SLE	0.89 (0.21–3.82)	0.087^b^
Global test	–	0.082
Sex		
Female	1	
Male	3.0 (1.20–7.50)	0.019^b^
Younger age at SLE diagnosis (per 10 years)	1.45 (1.01–2.07)	0.044^b^
Race		
Caucasian	1	
African	4.59 (1.79–11.76)	0.002^b^
Asian and other	0.93 (0.24–3.64)	0.913^b^
Hispanic	1.37 (0.39–4.85)	0.622^b^
Global test	–	0.005

a HR = hazard ratio; 95% CI = 95% confidence interval; NP = neuropsychiatric; SLE = systemic lupus erythematosus.

b By Wald's test.

### Treatment of SLE psychosis

The treatment of individual patients was at the discretion of their attending rheumatologists and was predicated on the overall needs of the patient and not only on the psychotic event. The following therapies were used during the time of the first psychotic events: corticosteroids in 23 of 28 patients (82.1%) with a mean ± SD dose of prednisone of 21.9 ± 14.9 mg/day, immunosuppressants (cyclophosphamide, azathioprine, methotrexate, mycophenolate mofetil) in 17 of 28 patients (60.7%), biologic agents in 1 of 28 patients (3.6%), antipsychotic drugs in 19 of 28 patients (67.9%), antidepressants in 11 of 28 patients (39.3%), and either/both antipsychotic drugs and antidepressants in 22 of 28 patients (78.6%). In 13 of 28 patients (46.4%), corticosteroids had been started prior to the onset of psychosis with a mean ± SD dose of 20.3 ± 13.6 mg/day.

### Clinical outcome and health‐related quality of life (HRQoL) in patients with lupus psychosis

**Figure 2:**
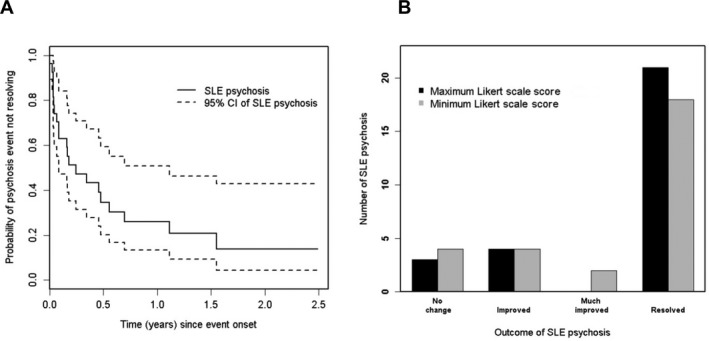
Physician‐determined outcome of lupus psychosis. **A,** Survival curve for resolution. **B,** Likert scale scores. The highest and lowest scores over the duration of follow‐up are shifted to the right, indicating improvement. SLE = systemic lupus erythematosus; 95% CI = 95% confidence interval.

A summary of physician assessments of outcome of lupus psychosis is illustrated in Figure 2. More than 80% of the psychotic events had resolved by the second annual assessment following onset of the event (Figure 2A). Likewise, the maximum and minimum Likert scores over the duration of follow‐up illustrate that the majority of psychotic events either improved or resolved during the period of observation (Figure 2B).

**Figure 3:**
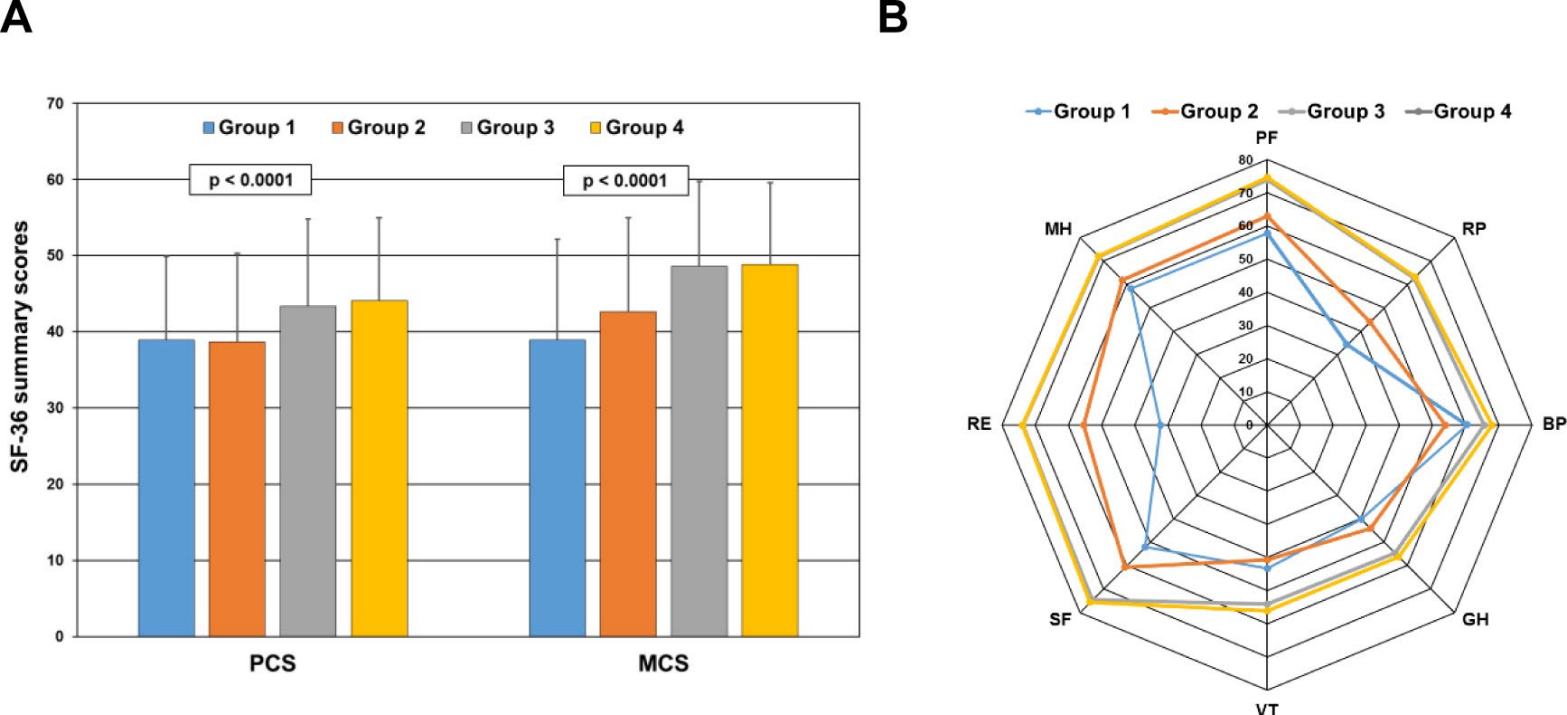
Association of Short Form 36 (SF‐36) summary and subscale scores with lupus psychosis. **A,** Mean ± SD physical component summary (PCS) and mental component summary (MCS) scores in 4 groups of patients with systemic lupus erythematosus (SLE). Group 1 consisted of patients with onset of lupus psychosis since last assessment or with an ongoing psychotic event (n = 29 visits). Group 2 consisted of patients with onset of other neuropsychiatric (NP) events since last assessment or ongoing other NP event(s), including non‐SLE psychosis (n = 3,379 visits). Group 3 consisted of patients with no NP events since last assessment and no ongoing NP event(s) but with a history of previous NP event(s) (n = 2,180 visits). Group 4 consisted of patients who never had NP event(s) (n = 5,893 visits). The numbers of assessments contributing to each bar are aggregated for patients over time. Global *P* values in the multivariate analyses are shown. **B,** Comparison of individual subscale scores in the same 4 patient groups. SF‐36 subscales are as follows: PF = physical function; RP = role physical; BP = bodily pain; GH = general health; VT = vitality; SF = social function; RE = role emotion; MH = mental health.

The mean ± SD SF‐36 PCS and MCS scores are shown in Figure 3A for 4 patient groups. Group 1 consisted of patients with onset of lupus psychosis since last assessment or with an ongoing psychotic event (n = 29 visits). Group 2 consisted of patients with onset of other NP events since last assessment or ongoing other NP event(s), including non‐SLE psychosis (n = 3,379 visits). Group 3 consisted of patients with no NP events since last assessment and no ongoing NP event(s) but with a history of previous NP event(s) (n = 2,180 visits). Group 4 consisted of patients who never had NP event(s) (n = 5,893 visits). The lowest summary scores were in groups 1 and 2 (global *P* < 0.0001 in the multivariate analyses), and the negative impact on HRQoL affected all 8 subscales of the SF‐36 as shown in the accompanying spidergram (Figure 3B).

**Figure 4:**
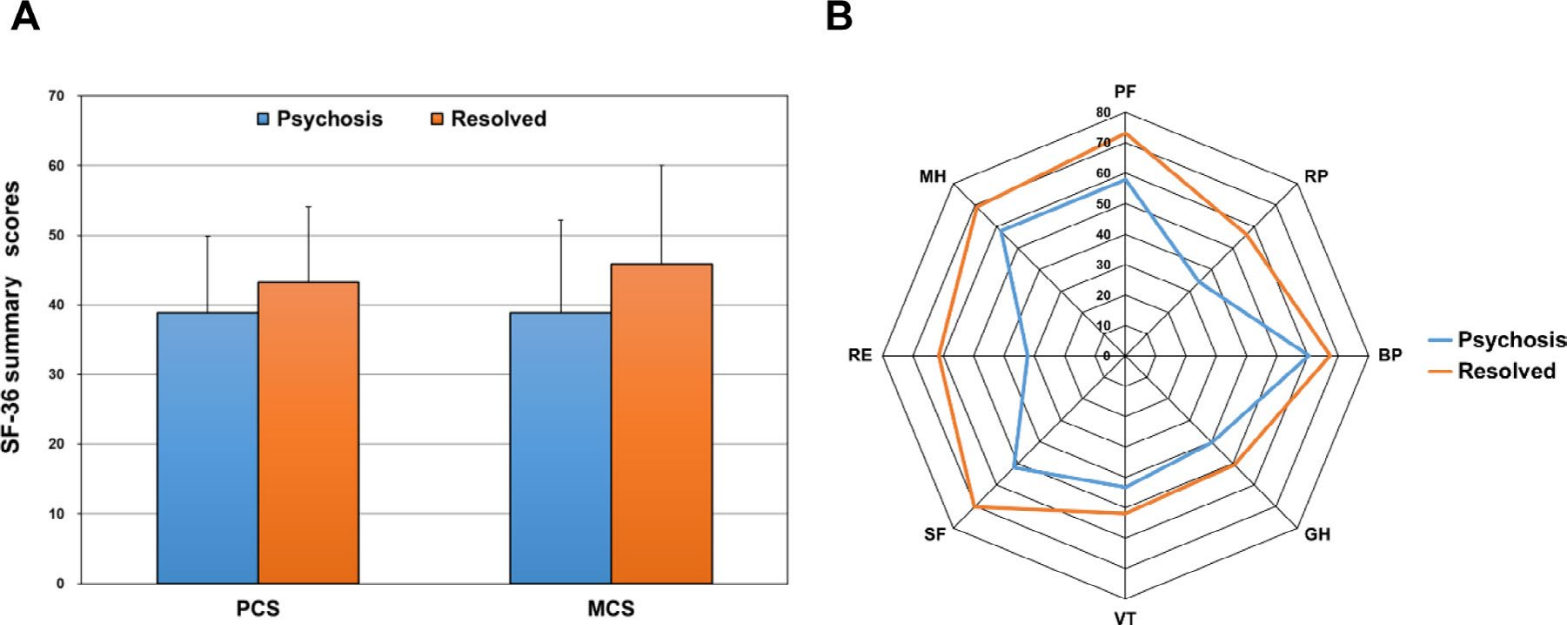
Long‐term change in Short Form 36 (SF‐36) summary and subscale scores following resolution of lupus psychosis. **A,** Mean ± SD physical component summary (PCS) and mental component summary (MCS) scores in 2 groups of patients with systemic lupus erythematosus. The psychosis group consisted of patients with onset of lupus psychosis since last assessment up to its resolution (n = 29 visits). The resolved group consisted of patients with resolution of lupus psychosis up to their last follow‐up visit or recurrence of psychosis (n = 112 visits). If the psychotic event had both onset and resolution in the same interval prior to assessment, SF‐36 scores at that assessment were included only in the psychosis group. The numbers of assessments contributing to each bar are aggregated for patients over time. **B,** Comparison of individual subscale scores in the same 2 patient groups. SF‐36 subscales are as follows: PF = physical function; RP = role physical; BP = bodily pain; GH = general health; VT = vitality; SF = social function; RE = role emotion; MH = mental health.

To determine if there was a persistent change in HRQoL following physician‐determined resolution of lupus psychosis, patient‐generated SF‐36 scores were compared in 2 groups. The psychosis group consisted of patients with onset of lupus psychosis since last assessment up to its resolution (n = 29 visits). The resolved group consisted of patients with resolution of lupus psychosis up to their last follow‐up visit or recurrence of psychosis (n = 112 visits). If the psychotic event had both onset and resolution in the same interval prior to assessment, SF‐36 scores at that assessment were included only in the psychosis group. As illustrated in Figure 4A, there was substantial improvement both in MCS scores (mean difference 7.01) and in PCS scores (mean difference 4.34) and in all subscales of the SF‐36 (Figure 4B) concurrent with resolution of lupus psychosis.

## Discussion

In a large international inception cohort study of SLE patients, we have prospectively documented the frequency, associations, and outcomes of psychotic events during a mean follow‐up period of 7.4 years. Our findings confirm and expand upon the results of previous cross‐sectional and historical studies of psychosis in SLE 6, 7, 8, 24, 25. The majority of psychotic events were directly attributed to SLE, had a tendency to occur early in the course of the disease, and were more frequent in male patients. Psychosis was also more frequent in patients of African ancestry, as is also the case for non‐SLE patients with the same race/ethnicity 26. The outcome of lupus psychosis, as determined by both physicians and patients, was positive and emphasizes the importance of diagnosing and treating this rare manifestation of NPSLE.

Studies of NPSLE conducted prior to the introduction in 1999 of the ACR case definitions for NPSLE did not have a uniform definition for psychosis. Using the ACR case definition, the frequency of psychosis has been reported to vary between 0% and 17.1% 6, 17, 27, 28, 29, 30, and in our study it was 1.53% (28 of 1,826 patients). Using a well‐defined process for determining attribution, we confirmed that the majority of psychotic events were due to SLE. In keeping with other NPSLE events and with other severe SLE manifestations such as nephritis 31, there was a tendency for psychosis to occur early in the disease course, usually within the first 3 years following the diagnosis of SLE. Univariate analysis identified significant associations between lupus psychosis and anti‐P antibodies, although following adjustment for demographic variables, the 95% CIs around HRs were wide and included the null value, precluding a definitive conclusion regarding association of these autoantibodies with psychosis. This is consistent with an earlier report on NP events in the SLICC inception cohort 32.

The potential role of corticosteroids must also be considered. In the current study, exposure to corticosteroids prior to lupus psychosis occurred in less than half of the initial events. As per the ACR case definition for psychosis 14, the concurrent use of corticosteroids at the onset of psychosis was identified as an association rather than as a firm exclusion, indicating uncertainty about the role of corticosteroids in individual cases and to allow flexibility for determining attribution. Although NP symptoms have been reported with all types and doses of corticosteroids 33, including psychosis following intraarticular steroid injections 34, 35, in general the dose of corticosteroids is the most important risk factor. In the Boston Collaborative Drug Surveillance Program (36), the frequency of psychiatric symptoms of any type was 18.6% in patients receiving >80 mg/day of prednisone, 4.6% in patients receiving 40–80 mg/day, and 1.3% in those receiving <40 mg/day. In the current study, exposure to corticosteroids prior to lupus psychosis was in the lowest of these dose ranges.

Although the somatic toxicities of corticosteroids are well described, the literature on NP effects is sparse. Their reported frequency varies widely from 2% to 60% 36, 37, 38, and symptoms include affective, behavioral, and cognitive manifestations 33. Moreover, the term “steroid psychosis” has been used to capture a heterogeneous group of NP effects and is not supported by validated diagnostic criteria, and previous studies have included many patients who were not psychotic. The ACR case definition for psychosis 14, used in the current study, is based on the Diagnostic and Statistical Manual of Mental Disorders, Fourth Edition (DSM‐IV) 39. In a previous study of 2,069 patients who received corticosteroids, only 3 (0.14%) developed psychosis according to DSM‐IV criteria 40.

One of the major advantages of our prospective study was the ability to document the short‐term impact and long‐term outcome of lupus psychosis from the perspectives of both the physician and the patient. In keeping with previous studies 6, 7, the physician assessments indicated resolution in the majority of cases with very few recurrences. Using a previously validated approach to measure the clinical outcome of NP events in SLE 18, we used summary and subscale scores of the SF‐36 to assess the patient perspective. This is important because physician and patient assessment of outcome for other manifestations of SLE 41 and some NP events 42 may be discrepant. Although the greatest impact was on MCS scores, it was apparent that all subscales of the SF‐36 were negatively impacted in patients with lupus psychosis. However, following treatment and in keeping with physician assessment of outcome, the patient‐generated SF‐36 scores showed a remarkable reversal when averaged over time.

There are some limitations to the current study. First, the small number of patients with lupus psychosis limited our ability to precisely estimate potential associations with clinical or laboratory variables of interest. However, most of the previous studies have had an even smaller sample size, and the SLICC cohort is the largest inception cohort of SLE patients. Second, specialized investigations such as advanced neuroimaging or cytokine profiling of cerebrospinal fluid were not routinely performed but were left to the discretion of individual investigators, which reflects what is done in clinical practice, a key component of our overall SLICC protocol. Third, the observational cohort study design precludes determination of optimal therapeutic regimens for lupus psychosis but instead reflects current standard of care. Despite these limitations, the study provides encouraging data on the outcome of this rare but potentially devastating manifestation of NPSLE.

## Author Contributions

All authors were involved in drafting the article or revising it critically for important intellectual content, and all authors approved the final version to be published. Dr. Hanly had full access to all of the data in the study and takes responsibility for the integrity of the data and the accuracy of the data analysis.

### Study conception and design.

Hanly, Urowitz, Gordon, Romero‐Diaz, Clarke, Merrill, Fortin, Gladman, Bruce, Petri, Zoma, Nived, Khamashta, Alarcón, Askanase, Farewell.

### Acquisition of data.

Hanly, Urowitz, Gordon, Bae, Romero‐Diaz, Sanchez‐Guerrero, Bernatsky, Clarke, Wallace, Isenberg, Rahman, Merrill, Fortin, Gladman, Bruce, Petri, Ginzler, Dooley, Steinsson, Ramsey‐Goldman, Zoma, Manzi, Nived, Khamashta, Alarcón, van Vollenhoven, Aranow, Mackay, Ruiz‐Irastorza, Ramos‐Casals, Lim, Inanc, Kalunian, Jacobsen, Peschken, Kamen, Askanase, Theriault.

### Analysis and interpretation of data.

Hanly, Li, Su, Urowitz, Gordon, Romero‐Diaz, Bernatsky, Clarke, Wallace, Fortin, Bruce, Zoma, Jonsen, Khamashta, Alarcón, van Vollenhoven, Inanc, Askanase, Farewell.
